# Traumatic Endophthalmitis due to *Cellulosimicrobium cellulans*

**DOI:** 10.1155/2011/469607

**Published:** 2012-01-30

**Authors:** Pimkwan Jaru-ampornpan, Anita Agarwal, Narinder K. Midha, Stephen J. Kim

**Affiliations:** ^1^Department of Ophthalmology, Vanderbilt Eye Institute, Nashville, TN 37232, USA; ^2^Clinical Microbiology Laboratory, Vanderbilt University School of Medicine, Nashville, TN 37232, USA

## Abstract

*Purpose*. To report a case of traumatic endophthalmitis due to *Cellulosimicrobium cellulans*. *Design*. Case report. *Methods*. Retrospective chart review. *Results*. To our knowledge, this is the first report of traumatic endophthalmitis due to *C. cellulans*, which did not respond to intravitreal antibiotics or repeat vitrectomy and ultimately led to the discovery of an occult intraocular foreign body. *Conclusions*. *C. cellulans* is a rare cause of endophthalmitis. Greater awareness of this bacterium in the ophthalmic literature as a cause of endophthalmitis and its association with foreign bodies may allow earlier and more purposeful intervention in future cases.

*Cellulosimicrobium cellulans*, formerly known as *Cellulomonas cellulans* or *Oerskovia xanthineolytica*, is a gram-positive rod that belongs to the Actinomycetales order [1]. It is found in soil and decaying plant material. It is relatively avirulent and until recently not known to cause ocular infection [2]. To our knowledge, we present the first case of traumatic endophthalmitis due to *C. cellulans, *which did not respond to intravitreal antibiotics or repeat vitrectomy. 

A 28-year-old healthy male was struck in his left (OS) eye by gravel while mowing his lawn but denied vision loss. Two days later, while fishing in deep water, he reported the sudden onset of pain and decreased vision OS. He presented the next day to an optometrist with visual acuity (VA) of 20/400 OS, severe anterior chamber inflammation, fibrin, and conjunctival injection. Intraocular pressure was 10 mmHg. Hourly topical prednisolone acetate 1% and oral methylprednisolone dose pack did not improve the inflammation and his condition worsened.

He was referred to the Vanderbilt Eye Institute 1 week after his injury. VA was hand motion (HM) OS, and intraocular pressure was 18 mmHg. Slit-lamp examination revealed a hypopyon with fibrin (Figure 1). Detailed examination of the globe revealed no obvious entry site wound. Funduscopic examination was limited by dense vitreous opacities, but ultrasound showed no signs of retinal detachment or intraocular foreign body (Figure 2). A vitreous biopsy with intravitreal injection of clindamycin 1 mg, vancomycin 1 mg, and ceftazidime 2 mg was performed. Testing for syphilis, lyme disease, toxoplasmosis, and toxocariasis was negative. Twenty-four hours later, VA worsened to light perception, and his culture results demonstrated moderate growth of gram-positive rods. He underwent immediate vitrectomy and intravitreal injection of vancomycin 1 mg, ceftazidime 2 mg, and moxifloxacin 160 *μ*g and started 60 mg of prednisone a day. VA improved to HM after surgery but then again worsened. Repeat vitrectomy and lensectomy was performed 12 days later and VA improved to 1/200. Repeat bacterial and fungal cultures were negative. The inflammation persisted and a subretinal, raised lesion could now be seen temporal to the macula. After > 2 weeks, the cultured bacterium was identified as *C. cellulans*. A third vitrectomy with scleral buckle was performed 9 days after the second vitrectomy because of worsening inflammation, decreasing vision, and exudative retinal detachment. The inflammatory lesion was unroofed and biopsied, and a small metallic wire (0.3 × 0.1 × 0.1 cm) was discovered and removed. Postoperatively, the inflammation resolved and VA improved to 20/70. An orbital CT scan confirmed no remaining intraocular foreign body (Figure 3). 

Until recently, *C. cellulans *had not been reported as a cause of ocular infection, but 3 clustered cases of postoperative *C. cellulans *endophthalmitis from presumed contamination were reported in 2010 [2]. To our knowledge, however, this is the first case of traumatic endophthalmitis due to *C. cellulans,* and this case highlights several important features about its presentation. There was a substantial delay in identifying the bacterium by our laboratory because of its rarity and lack of previous association with endophthalmitis. *C. cellulans* is an uncommon but increasingly recognized cause of human infection and generally occurs in the setting of immunodeficiency or foreign bodies [3]. Antibiotic sensitivity testing using the Kirby-Bauer disk diffusion method demonstrated complete resistance of the organism to ceftazidime, but sensitivity to vancomycin, moxifloxacin, and amikacin. Had *C. cellulans *been identified more promptly in this case and its association with foreign bodies and endophthalmitis known to us, we would have immediately focused our attention on an occult intraocular foreign body, which would have connected the antecedent events of this case. Such invaluable insight may have allowed us to remove the source of the recurrent inflammation sooner as opposed to relying on events that unfolded.

In summary, we present, to our knowledge the first case of *C. cellulans* traumatic endophthalmitis. Greater awareness of this bacterium in the ophthalmic literature as a cause of endophthalmitis and its association with foreign bodies may allow earlier and more purposeful intervention in future cases.

## Figures and Tables

**Figure 1:**
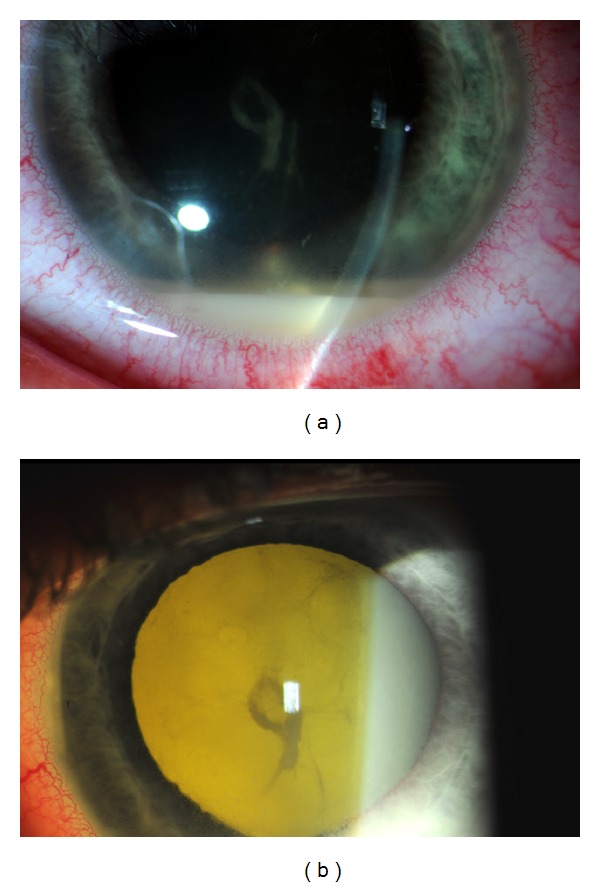
External photograph on initial presentation to Vanderbilt illustrating hypopyon (a) and retroillumination demonstrating dense vitreous opacities (b).

**Figure 2:**
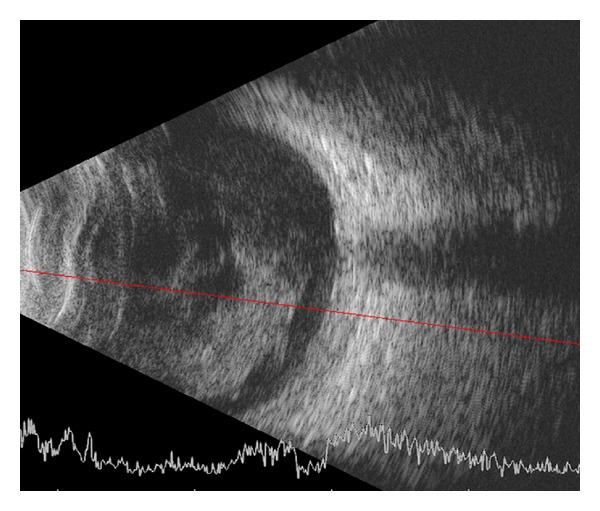
B-scan ultrasound demonstrating no signs of retinal detachment or obvious intraocular foreign body.

**Figure 3:**
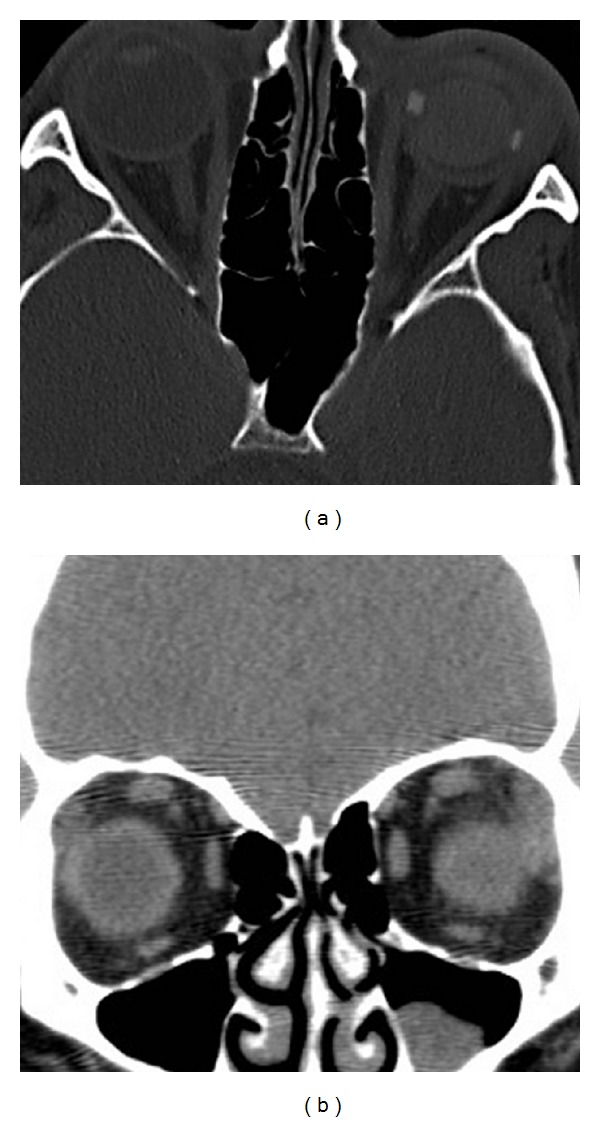
Orbital CT scan showing no residual intraocular foreign body.
